# Workplace social capital and mental health: a cross-sectional study among Iranian workers

**DOI:** 10.1186/s12889-018-5659-3

**Published:** 2018-06-26

**Authors:** Mojgan Firouzbakht, Aram Tirgar, Tuula Oksanen, Ichiro Kawachi, Karimollah Hajian-Tilaki, Maryam Nikpour, Susan Mouodi, Reza Sadeghian

**Affiliations:** 1 Social Determinants of Health Research Center, Health Research Institute, Babol University of Medical Sciences, Babol, Iran; 2 Finnish Institute of Occupational Health, Turku, Finland; 3 Department of Social and Behavioral Sciences, Harvard T.H. Chan School of Public Health, Boston, MA USA; 4 Department of Biostatistics and Epidemiology, Babol University of Medical Science, Babol, Iran; 5 Babol, Iran

**Keywords:** Social capital, Workplace, Mental health, Workers, Iranian, Psychological distress

## Abstract

**Background:**

The psychosocial environment of the workplace has received less attention in terms of occupational health. Trust, social network and social cohesion at the workplace (that is, factors related to social capital) may have effects on employee health. Thus, the objective of this study was to examine the association between workplace social capital and mental health among Iranian workers.

**Methods:**

In this cross-sectional study, data were obtained from 5 factories in Babol, Northern Iran, in 2016, where 280 workers responded to a survey on social capital at work and psychosocial distress.

**Results:**

Approximately 23.6% of the workers had psychological distress, and 23.4% had low social capital in the workplace. There was a significant relationship between mental health and individual workplace social capital (*p* = 0.025) and aggregated workplace social capital (*p* = 0.027). After controlling for each individual’s characteristics, the prevalence ratio of psychological distress was 2.11 (95% CI: 1.43-3.17) times higher among workers with low individual social capital, and low aggregated workplace social capital was associated with 2.64 (95% CI: 1.28–5.45) times higher odds of psychological distress.

**Conclusion:**

Higher social capital is associated with a reduced risk of psychological distress. The promotion of social capital can be considered as a means to increase workplace mental health among workers.

## Background

In developing countries, a wide range of diseases (including physical and mental diseases) are associated with social factors [1]. The WHO has further stated that social capital is one of the factors affecting health that could be a missing link in health-related studies [2], be protective of mental health and reduce stress [3]. Social capital is defined as the resources accessed through social networks [4, 5]. These resources include the exchange of tangible support (e.g., cash loans, labor in kind, etc.) between network members, as well as intangible resources such as emotional support or the diffusion of information. Social capital is hypothesized to promote health through several mechanisms including the following: (a) strengthening the individual’s ability to cope with stress (the “buffering hypothesis”), (b) acting in accordance with the established norms among the individuals within a group or workforce, and (c) boosting the individuals’ ability to participate in collective action to guarantee its members’ benefits (“collective efficacy”). Social capital has been studied in a number of settings, including the family [6], neighborhood [7] and the workplace [8–10]. Considerable efforts have been applied in recent years on understanding the role of workplace social capital as a determinant of workers’ health. The workplace is considered to be a major social “context” to which working age adults devote a large fraction of their waking time [11]. Employment status as well as specific conditions in the workplace, such as occupational stress, working hours and job insecurity, has been shown to have a large influence on employee mental health [12]. In the past few decades, the psychosocial environment of the workplace as a determinant of employee health has received much attention. According to some investigations, characteristics of workplace social capital, namely, trust, social networks and social cohesion, probably influence employees’ health [8, 13]. Organizations with high social capital have more satisfied, healthier, happier and more productive employees than those with low social capital [14]. A cohort study among 48,592 workers in the public sector in Finland showed that low social capital is related to a 20–50% increased risk for onset of depression [14]. A study on 2000 industrial workers the USA showed that low social capital is related to reduced smoking and job stress [3]. However, there is a lack of evidence for the effect of social capital at work on mental health in developing countries [10].

Therefore, a cross-sectional study was conducted to evaluate the workplace social capital in five factories in Iran. Both individual and workplace level social capital and their association with employee mental health were assessed using a psychological distress questionnaire.

## Methods

### Study population and design

A cross-sectional study was performed at five factories (26 units nested within factories) in the city of Babol on the coast of the Caspian Sea in Northern Iran in 2016. The factories were selected using convenience sampling from a variety of industries: the metal (*n* = 250), cellulosic products (*n* = 200), foodstuffs (*n* = 80), and electrical and home appliance (*n* = 150) industries. The sample size in the study was estimated to detect the effect size of 0.25 with a 95% confidence interval and 80% power of 240 subjects with estimated 20% dropped samples of 285 employees. Thus, questionnaires were administered to 350 randomly selected workers who met the inclusion criteria, and 280 workers responded (response rate of 81%).

The inclusion criteria included at least 6 months work experience in the factories. Employees with a history of mental illness at the onset of work, or that had a stressful event (divorce, marriage, death of close relative, etc.) within the last 6 months were excluded. Survey data on demographic characteristics, social capital at the workplace and mental health were obtained.

### Mental health

Mental health was assessed using the General Health Questionnaire, GHQ-12. This is a brief, simple and valid tool for measuring psychological distress [15]. This questionnaire consists of 12 questions rated on a four-point Likert scale (less than usual, not more than usual, rather more than usual, or much more than usual). The first two options were given a 0 score and the other two options a 1 score; the range of total scores in this questionnaire is 0–12. The cut-off point of 3.5 was used in this questionnaire and higher scores indicated mental health problems [16]. This questionnaire has been translated to different languages, including Persian [17], and has been validated in several studies in Iran [16].

### Workplace social capital

Social capital in the workplace was assessed using an 8-item questionnaire designed and validated in the Finnish Public Sector study by Kouvonen et al. [9]. Reliability of the questionnaire has been confirmed in several studies with a Cronbach’s alpha coefficient of 0.88 [18]. The responses were given on a 5-point Likert scale: from 1 (totally disagree) to 5 (totally agree); the total scores ranging between 8 and 40 and an average score [23] was used for dichotomizing low and high social capital, using the mean score of cut off values. A higher score indicated a high level of social capital. Social capital was assessed at the individual and workplace levels using this tool. The aggregate workplace social capital was calculated using the scores of at least three coworkers in the same work unit [9].

### Data were obtained from 26 units in the 5 factories

The questionnaire was translated using the International Quality Of Life (IQOLA) proposed protocol with backward and forward translations techniques [19].

After obtaining permission from the authors, it was translated into Persian by two independent translators. After examining the semantic equivalents, the difficulty and clarity of the two translations were mixed and the Persian questionnaire was returned to two other translators to restore the original context (backward and forward translation).

The last stage was also repeated in this process. Then, the questionnaire was sent to the authors for approval. The validity was evaluated in 30 eligible workers in a pilot study.

The internal consistency was examined using Cronbach’s alpha. In addition, the test retest consistency was examined with a two-week interval, and the intra-class correlation (ICC) was estimated. The acceptable minimum correlation coefficient was 70%. The results of the tests were: test-retest correlation, 0.68; Cronbach’s alpha, 0.78 and ICC, 0.76 (95% CI 0.38–0.85).

### Covariates

The demographic characteristics included the social and individual characteristics (age, sex, marital status (single/married), education level (high school/university), economic status (self-reported poor, moderate or good), work experience (years), hours of work per month, shift work (yes/no), and the number of coworkers in their own work unit.

Self-rated health was used to measure health. The respondents completed an assessment of their current health using a five-point Likert scale (1 = very good, 2 = good, 3 = average, 4 = poor and 5 = very poor). Self-rated health is one of the most widely used measures of health status [20], which is shown to be related to a number of important medical endpoints [21, 22] and sensitive to changes in health status [23]. In this study, the scale of self-rated health was dichotomized to “good” (responses 1–2) and “poor” (responses 3–5).

### Statistical analysis

The association between baseline characteristics and workplace social capital and mental health was analyzed using descriptive statistics. The log binomial regression was used to estimate the adjusted prevalence ratio (PR) and its 95% confidence interval for individual social capital data. For estimation of the aggregated workplace social capital and poor mental health, a generalized estimating equation (GEE) was used with an exchangeable correlation matrix and binary logistic model, for adjusted clustering of workers within work units. Missing data (3–10%) are presented in our data; to handle missing values in the calculation of the total score, the missing data were individually replaced by the mean value of scores. All statistical analysis was performed using the SPSS statistical software version 21 (Chicago,Illinois: SPSS Inc.) with a significance level of less than 0.05, and all tests were performed for two sides.

## Results

The mean age (SD) and work experience of the workers were 32.2 (6.7) and 7.4 (5.3) years, respectively, and the mean monthly working hours was 230.98 (60.6) (Table 1). The mean (SD) score of individual level social capital was 31 (5.9) and 23.6% of the workers have psychological distress. Table 2 shows the associations of the baseline covariates and social capital and mental health. For example, economic status was associated with mental health (*P* = 0.004); education was associated with individual workplace social capital (*p* = 0.012) and aggregate workplace social capital (*p* = 0.01). Self-reported health was associated with individual level workplace social capital (*p* = 0.001((Table 2).

**Table 1:** Demographic characteristics of Iranian workers

Demographic Characteristic		N(%)/Mean±SD( Median)
Age (year)		32.21 ± 6.71 (31)
Work experience(year)		7.46 ± 5.27 (7)
Time of working (hours/month)		230.98 ± 60.6 (230)
Gender	Women	78(29.3)
	Men	188(7o.7)
Marital Status	Single	57(23.2)
	Married	188(76.8)
Education	High school	76(28.1)
	University	194(71.9)
Shift work	Yes	166(64.09)
	No	93(35.9)
Residential status	City	143(58.3)
	Village	102(41.7)
Economic Status	Poor	89(33.3)
	Moderate	144(53.9)
	Good	34(12.7)
Self- health rated	Poor	116(43.9)
	good	148(56.1)

**Table 2:** Relationship between demographic characteristics and mental health, workplace social capital (individual and aggregation) of worker

Demographic Characteristic		Mental Health		P	Workplace social capital					
					Individual		P	Aggregated		P
		Yes N(%)	No N(%)		High N(%)	Low N(%)		High N(%)	Low N(%)	
Gender	Women	53(27.2)	24(20.9)	NS	58(24.6)	13(21.1)	NS	44(24.2)	14(14.5)	NS
	Men	125(72.8)	63(79.1)		152(75.4)	38(78.9)		133(57.8)	63(58.5)	
Marital Status	Single	37(17.1)	20(14.7)	NS	39(14.7)	21(28.9)	NS	17(11.3)	39(17.8)	NS
	Married	131(82.9)	58(85.3)		178(85.3)	37(71.1)		65(88.7)	144(82.2)	
Education	High school	51(29.1)	25(35.8)	NS	70(32.5)	11(21.1)	NS	60(36.8)	19(31.1)	0.01*
	University	132(70.8)	62(62.2)		145(67.3)	47(78.9)		160(63.2)	31 [6]	
Shift work	Yes	112(65.8)	54(69.7)	NS	128(70.3)	34(70.3)	NS	101(63.5)	47(75.8)	NS
	No	63(34.1)	30(30.3)		81(29.7)	19(29.7)		58(36.4)	15(23.2)	
Residential status	City	103(61.7)	40(50.7)	NS	26(47.4)	28(52.6)	NS	120(69.6)	34(44.1)	0.001*
	Village	67(83.3)	42(49.3)		81(39.5)	123(60.5)		57(30.4)	41(55.9)	
Economic Status	Poor	51(27.6)	38(4.5)	0.008*	22(43.6)	15(4.5)	NS	64(62.1)	28(27.1)	NS
	Moderate	107(63.5)	37(86.4)		23(46.2)	175(86.4)		106(58.8)	37(50)	
	Good	21(8.8)	13(7.6)		10(10.3)	25(7.6)		16(6.1)	14(12.9)	
Self- health rated	Poor	77(38.4)	39(58.9)	0*	83(41.9)	32(50)	0.001*	73(40.5)	27(34.6)	NS
	Good	108(16.7)	38(41.2)		123(48.1)	32(50)		107(59.4)	51(65.3)	

*Significance level = *p* < 0.05, NS=No significance

A statistically significant relationship was found between mental health and individual level social capital. The PR low versus high workplace social capital on low mental health was 2.11 (95% CI: 1.43–3.17) when adjusted for all baseline covariates. Similarly, low aggregated workplace social capital was associated with poor mental health after adjusting for clusters: OR = 2.64 (95% CI: 1.28–5.45) (Table 3).

**Table 3:** Crude and adjusted PR (CI 95%) and OR (CI 95%) association of individual and aggregated workplace social capital and poor mental health

		Unadjusted Model	Adjusted model
Individual workplace social capital(PR)	high	1(−)	1(−)
	low	1.68(1.05–2.69)	2.11(1.41–3.17)
Work place social capital(OR) Aggregated	High	1(−)	1(−)
	low	2.43(1.27–4.67)	2.64(1.28–5.45)

## Discussion

This study aimed to evaluate the associations between workplace social capital and mental health among Iranian workers. It had been found that both low individual and aggregated workplace social capital were associated with poor mental health. Several studies have shown the relationship between mental health and social capital [11, 14, 24, 25]. The results of the present study are in line with those of Patussi [24] in Brazil; they showed that an increase in social capital in female workers was associated with an improvement in mental health and the promotion of healthy behaviors.

Social capital and mental health may be connected by several potential mechanisms including the individuals’ ability to address stress [3, 25], and occupational stress [26, 27] increases if there is higher social capital in the workplace. Second, the support of fellow workers is beneficial to the employees [28] because social support is a source of health promotion and provision [29]. Third, in an integrated workplace, health behaviors and collective actions can be more efficient and can reduce occupational injuries in the workplace [28, 29]. Fourth, healthy behaviors and collective action are believed to be more effective in integrated workplaces and reduce job-related injuries in the workplace [28, 29]. Moreover, health related information is probably circulated more rapidly [30] in work environments with higher social capital [28]. It was shown by some investigators that the psychological distress of the employees is impeded by the buffering effects of their workplace social capital [31].

The results of this study showed that 23.6% of workers had psychosocial distress. These results are consistent with reports by other researchers [32–34]. In the current study, economic status was associated with mental health. The finding corresponds with previous studies showing that financial stress (low income or poverty) and economic insecurity (unemployment or temporary jobs and debts) increase the risk of mental distress [35]. Moreover, in health inequality studies, the social environment is a social factor that can improve health. Social capital may buffer the inequality of health [36]. People who have higher social capital and higher social support have more access to material (money, food and health centers) and nonmaterial (information and health-related norms) resources [37].

Several studies indicated that social capital at the individual level can be determined by various socio-economic factors such as educational level, economic status and employment status [26, 38–40]. In other words, social capital is most likely to be determined by the social context in which an individual lives, such as family, neighborhood and work environment [41]. Conceptually, social capital reflects the social structure of relationships and is a collective feature [42]. In this study, workplace social capital was associated with the level of education and number of coworkers, which is similar to the results of Oksanen [14], who reported a significant correlation between the characteristics of the workplace (number of coworkers, hours of work absences and manual jobs) and workplace social capital.

The results of this study need to be interpreted in light of the following strengths and limitations. This is the first study on workplace social capital and health in the Iranian workforce. Although the sample size is small, the response rate was sufficient and workplace social capital was assessed with a standardized workplace social capital questionnaire.

Workplace social capital may be affected by social capital outside workplaces, and vice versa. However, social capital outside the workplace setting was not assessed. This study is cross-sectional, and thus, the temporal sequence of low workplace social capital and poor mental health remained unsolved. Thus, causal interpretation of its apparent association must be done cautiously.

## Conclusion

The result of this study shows that high social capital in the workplace is associated with a better mental health status of workers. Therefore, the promotion of social capital can be introduced as one of the strategies to improve mental health in the workplace. Additional studies on social capital and mental health in developing countries are needed.

## Abbreviations

CI: Confidence interval
GEE: Generalized estimating equation
GHQ: General health questionnaire
ICC: Intra-class correlation
IQOLA: International quality of life assessment
OR: Odds ratio
PR: Prevalence rate
WHO: World Health Organization
